# Fused Filament Fabrication and Computer Numerical Control Milling in Cultural Heritage Conservation

**DOI:** 10.3390/ma16083038

**Published:** 2023-04-12

**Authors:** Daniela Fico, Daniela Rizzo, Francesco Montagna, Carola Esposito Corcione

**Affiliations:** 1 Department of Engineering for Innovation, University of Salento, Edificio P, Campus Ecotekne, s.p. 6 Lecce-Monteroni, 73100 Lecce, Italy; 2 Department of Cultural Heritage, University of Salento, Via D. Birago 64, 73100 Lecce, Italy

**Keywords:** advanced technologies, cultural heritage, conservation, additive manufacturing, computer numerical control milling

## Abstract

This paper reports a comparison between the advantages and disadvantages of fused filament fabrication (FFF) and computer numerical control (CNC) milling, when applied to a specific case of conservation of cultural heritage: the reproduction of four missing columns of a 17th-century tabernacle. To make the replica prototypes, European pine wood (the original material) was used for CNC milling, while polyethylene terephthalate glycol (PETG) was used for FFF printing. Neat materials were chemically and structurally characterized (FTIR, XRD, DSC, contact angle measurement, colorimetry, and bending tests) before and after artificial aging, in order to study their durability. The comparison showed that although both materials are subject to a decrease in crystallinity (an increase in amorphous bands in XRD diffractograms) and mechanical performance with aging, these characteristics are less evident in PETG (E = 1.13 ± 0.01 GPa and σ = 60.20 ± 2.11 MPa after aging), which retains water repellent (ca = 95.96 ± 5.56°) and colorimetric (∆E = 2.6) properties. Furthermore, the increase in flexural strain (%) in pine wood, from 3.71 ± 0.03% to 4.11 ± 0.02%, makes it not suitable for purpose. Both techniques were then used to produce the same column, showing that for this specific application CNC milling is quicker than FFF, but, at the same time, it is also much more expensive and produces a huge amount of waste material compared to FFF printing. Based on these results, it was assessed that FFF is more suitable for the replication of the specific column. For this reason, only the 3D-printed PETG column was used for the subsequent conservative restoration.

## 1. Introduction

Additive Manufacturing (AM), first named rapid prototyping before becoming known as “3D printing”, is an innovative technology able to produce 3D models with complex shapes using a layer-by-layer building strategy. By contrast, in recognized traditional techniques, such as subtractive manufacturing (SM) and formative manufacturing (FM), the material is removed via machining, drilling, or grinding methods, or cast into molds [1]. Due to its fast expansion, the aim of AM has shifted from rapid prototyping to rapid tooling, and now to 3D manufacturing. AM has included several manufacturing techniques (photopolymerization, powder bed fusion, directed energy deposition, material extrusion, binder jetting, curing, lamination, etc.), to create a wide range of machineries of possible importance to industry. The driving force of the exponential increase in AM technologies could be attributed to the important results achieved by academic and industrial researchers in developing low-cost technologies and innovative high-tech materials [2,3,4,5,6,7,8]. Numerous aspects inform the selection of the most suitable manufacturing method for a specific application, such as cost, geometric complexity, material usage and properties, time, energy consumption, and sustainability. More details are reported in a previous review by the authors [9], where the classification of the most important AM techniques with a description of respective processing is reported. The advantages and disadvantages of these technologies are also reported. One of the most common problems lies with the instability of these devices, creating imperfections in the 3D printed models and differences in surface roughness across 3D reproductions with the same digital input [2]. SM techniques produce machined components with elevated accuracy and low geometrical complexity. AM allows for the creation of very complicated geometry with a lower tolerance and relative quality [10]. Several authors have proposed a solid analysis of the similarities, differences, advantages, and disadvantages of AM vs. SM through documented international works, based on these key factors. As an example, the Wohler’s 2013 Report presents a wide overview of AM’s incorporation into the industrial market [11], evidencing where AM can dominate SM, or otherwise cannot compete with conventional techniques. Referring to high-volume production, the high venture capital necessary to produce 3D models using AM does not make it a financially reasonable choice for producers [12]. Traditional techniques, such as, for example, injection molding, still appear more economical and suitable for this context, even if AM allows for the production of models with high geometrical complexity, removing, shaping, and linking the process by printing complete parts in a single step. An example of this is the metal acetabular cup [13] used in hip replacement surgeries; in the past it was produced using several SM and formative manufacturing processes, such as forging, machining, and coating. Contrastingly, an AM powder-bed fusion metal printer can build the acetabular cup and add the porous surface into the surface layers in a single print.

Another important field of application is mass customization, identified by its capability to offer exclusively designed products and services to each consumer because of elevated process agility, flexibility, and integration [14,15,16]. The lack of lead time and fast design modification in AM, together with exclusive representation supplied by 3D scanning, puts AM techniques ahead of SM processes for this specific application. Referring to the low-volume manufacture of products, which is defined specific to the industry, product, and sale capacity [17], AM manufacturers can achieve a superior complexity with equivalent costs. The launch of AM into the manufacturing sector started, in fact, with low-volume production for rapid prototyping with stereolithography. Until now, functional assembly manufacturing remains the main application of AM, as also reported by Wohler [11]. Another paper [17] compared the production cost of a small plastic bar made using AM powder-bed fusion with that of one made with injection molding, suggesting that for a production volume smaller than 10,000, AM had a reduced unit cost compared to injection molding [17]. While SM dominates the mass manufacturing region financially, AM is better suited to producing the tooling and fixtures necessary for conventional mass manufacturing molds [18,19]. AM presents a decreased lead time and cost, taking advantage of high value, low production of parts such as those used in ships, automation, aviation, and satellites, etc. AM also offers producers the capability to trade complexity for customizability in low volume production, where AM is being utilized for personally customized restoration components [20]. Nevertheless, AM machines offer production flexibility, and they are still significantly expensive in comparison to SM techniques. On the other hand, AM allows for a reduced production of wasted material and higher resource efficiency, which, importantly, has a good effect on the environmental impact, in comparison to the SM machines [4,5,9,21]. Strategic conditions for the diffusion of AM into the broader trade market involve high process stability, a database including AM material properties, on-line quality control processes, constant certification, and preparation of design guidelines. Nowadays, the growth of gradually more precise technologies, the reduction of costs, and the development of innovative high-tech materials allow for the successful use of AM techniques in several application fields, such as cultural heritage [22]. In a previous work [22], the authors demonstrated the suitability of the fused filament fabrication (FFF) method for the reproduction and restoration of four missing columns of a 17th-century polychrome wooden tabernacle belonging to a private collection. In this paper, the same columns were also reproduced with a traditional SM technique, with the aim of comparing the two different technologies in the same specific application.

## 2. The Case Study

**Figure 1:**
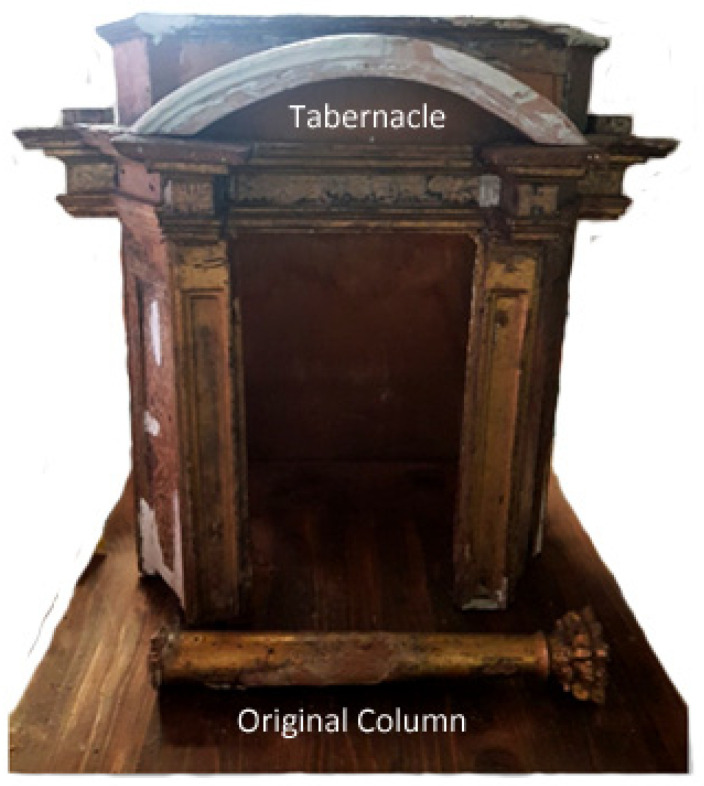
17th-century polychrome wooden tabernacle and original column.

This research aims to evaluate the general effectiveness of the two different methodologies in the restoration and conservation of artistic heritage; specifically, it assesses the advantages and disadvantages of each selected technique (fused filament fabrication (FFF) and computer numerical control (CNC) milling) in the reproduction of missing columns of an ancient 17th-century polychrome wooden tabernacle (Figure 1). The tabernacle is made of pine wood and according to oral sources it was handed down. With preliminary observation under the optical microscope [23], it can be seen to be decorated with an artistic technique called “Estofado de Oro”.

The experimental work carried out in this paper started with the characterization of the materials used in both CNC and FFF techniques, and the subsequent comparison of some of the properties that were most interesting for conservation purposes. Next, the ancient column was replicated by using PETG for FFF and pine wood for CNC. A deep comparison analysis of the times, costs, and waste production of the two techniques was also performed before the final restoration step.

**Figure 2:**
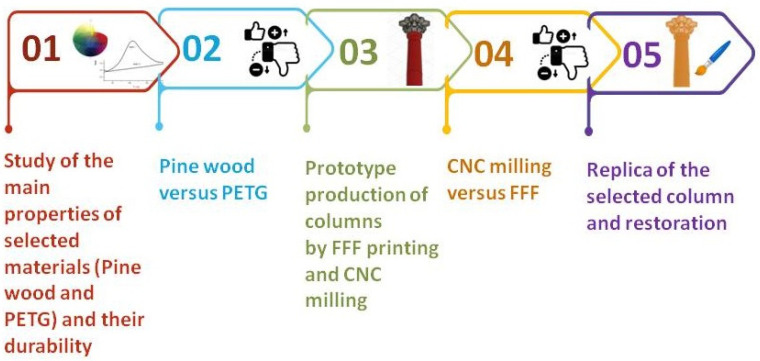
Experimental activity in summary.

The experimental activity consisted of the following steps, summarized in Figure 2.

## 3. Materials and Methods

### 3.1. Characterization of the Materials for FFF and CNC Milling Process

#### 3.1.1. Chemical Characterization of Neat Materials

Pine wood was selected to produce the column prototype using the CNC milling technique because it is similar to the original material of the tabernacle, as reported from oral sources and confirmed by preliminary optical microscope observations. The European pine wood used in this work was supplied by Tecno Wood SRL (Lecce, Italy) with the following features: light yellow colour, straight grain, medium texture, weight 2373.79 g, length 799 mm, and square cross-section with 79 mm side.

Polyethylene terephthalate glycol (PETG), supplied by the company PrimaSELECT (Malmo, Sweden), was used for the FFF printing of the columns. It was selected by comparing its properties (such as durability and thermal stability) to that of other materials usually used in 3D printing [22]. The PETG filament has a diameter of 1.75 ± 0.05 mm, a density of 1.27 g cm^−3^, and a melt flow index (MFI) of 12.1 g/10 min at a temperature of 225 °C, according to the supplier’s data sheet.

Fourier-transform infrared spectroscopy (FTIR) analyses were performed on the neat materials (pine wood and PETG) to carry out a preliminary chemical structural characterization. FTIR spectra were obtained on KBr pellets (1 mg of wood powder and PETG and 200 mg of KBr) using a JASCO FT/IR 6300 spectrometer (Easton, MD) with a resolution of 4 cm^−1^, setting up 64 scans in the region between 4000 and 600 cm^−1^. Five spectra were considered for each replicate sample.

#### 3.1.2. Durability Analysis

To study the durability of the materials used for the reproduction of the tabernacle columns, and to obtain more information on the average lifetime of the replicas, PETG and pine wood were subjected to accelerated aging tests.

The samples used for the accelerated aging test (bars with dimensions of 60 mm × 13.2 mm × 0.7 mm) were produced from pine wood and PETG, according to ISO178:2014. Specifically, the pine wood samples were produced by cutting the wooden stock piece manually, while the PETG samples were produced using the BQ HEPHESTOS 2 printer (BQ Company, Madrid, Spain) and setting the following operating conditions: extrusion temperature 220 °C, printing speed 50 mm/s, infill 20%. The CAD model was created with Fusion 360 software (Autodesk, San Rafael, CA, USA), converted to an STL file with Cura software (Ultimaker B.V., Utrecht, The Netherlands).

Artificial aging was carried out by placing the samples in Binder model KMF115 climatic chamber (BINDER GmbH, Tuttlingen, Germany) (T = 60 ± 2 °C, RH% = 80 ± 1%) and under a xenon-arc UV lamp for 5 days [24].

Morphological structural, thermal, and mechanical analyses were carried out on test samples, before and after exposure to artificial aging, to study the durability of the materials:

− Dynamic contact angle measurements were carried out with the First Ten Angstroms FTA1000 Quick Start instrument (Newark, CA, USA) equipped with a video camera, and analyses were performed at room temperature using the sessile drop technique, according to NORMAL-33/89.
− Evaluation of color change of materials before and after artificial aging was carried out with a Konica Minolta CR-410 (Milano, Italy), equipped with a Xenon lamp. Measurements were made following the recommendations of NORMAL-43/93 and using the CIELab International Chromatic System (1976). The color changes were evaluated by the L*a*b* system and expressed as ∆E.
− XRD measurements were carried out with a Rigaku Ultima+ diffractometer (Tokyo, Japan) with CuKα radiation (λ = 1.5418 Å) in the step scan mode recorded in the 2θ range of 5–60°, with a step size of 0.02° and a step duration of 0.5 s. For each replicate sample, three spectra were considered.
− DSC analysis (Mettler Toledo DSC1 StareSystem, Milano, Italy) was performed on 3D-printed PETG samples to investigate glass transition temperature (T_g_) variations, over a temperature range of 25 °C to 200 °C (heating rate of 10 °C/min).
− Flexural tests were performed on the samples using a Lloyd LR5K dynamometer (Lloyd Instruments Ltd., Bognor Regis, UK), with a test speed of 2 mm/min and a specimen support spacing of 64 mm, according to the ISO178(2014). For each sample, five replicates were made.

### 3.2. Replica of the Column by FFF Printing and CNC Milling

The CNC milling machine model HURCO VM 10 (Gindumac GmbH, Kaiserslautern, Germany) and the BQ HEPHESTOS 2 printer (BQ Company, Madrid, Spain) were used to reproduce the columns of the ancient tabernacle and to compare CNC milling versus FFF printing.

**Figure 3:**
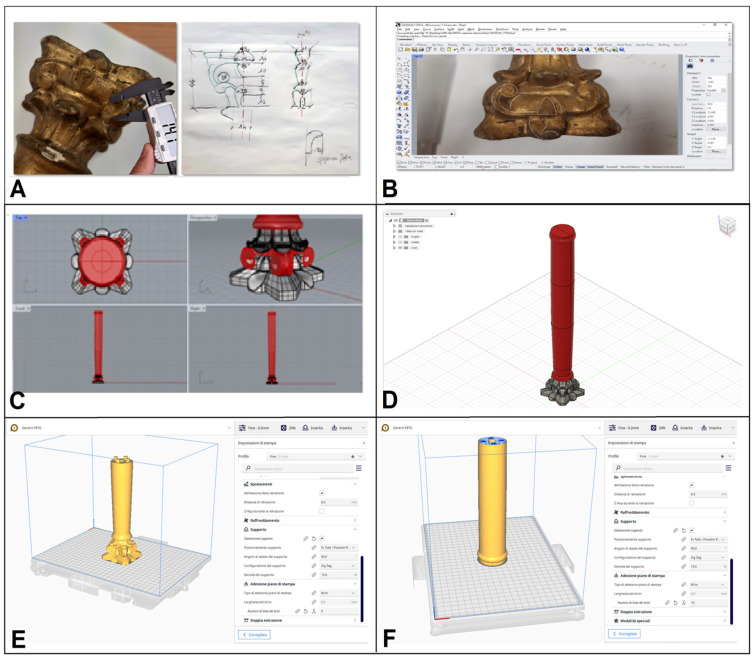
Surveying measurements with a caliper (left) and drawing by hand (right) (**A**); 2D drawing with Rhinoceros software **(B**); 3D modeling of the column using Rhinoceros software (**C**); CAD model modified with Fusion 360 software for CNC milling (**D**); CAD models modified with Cura software for 3D printing (**E**,**F**).

Specifically, the creation of the column replica with both methodologies followed the steps below (Figure 3):

− Morphological survey and manual drawing of the column (Figure 3A) [22];
− Transformation of the 2D model into CAD model using Rhinoceros software (Robert McNeel & Associates, Seattle, DC, USA), Figure 3B,C [22];
− Modification of the CAD model using Fusion 360 software (Autodesk, San Rafael, CA, USA); specifically, while a single CAD file was sufficient for the CNC milling technique (Figure 3D), for the 3D printing of the column it was necessary to separate the half-height 3D model into two parts and create two separate CAD files (base and capital, Figure 3E,F), due to the small print volume (210 mm × 297 mm × 220 mm) compared to the total height of the column (H 38.7 cm).
− Transformation of CAD files into G-Code and STL, using Cura software (Ultimaker B.V., Utrecht, The Netherlands);
− FFF printing of the PETG column using the following operating parameters of the 3DPRN LAB printer: 0.4 mm nozzle, 20% infill, extrusion temperature of 220 °C, platen temperature of 50 °C, printing speed of 60 mm/s, layer height of 0.2 mm;
− Reproduction of the pine wood column by CNC milling by setting the following operating parameters in the Fusion 360 software: HURCO VM 10 machine, pine wood material. Functions and cutters were set according to the different steps, as will be reported in the Section 4.2.

### 3.3. Materials for Restoration

The materials used for the restoration of columns produced were: Bologna gypsum (CTS SRL, Bari, Italy), rabbit glue (CTS SRL, Bari, Italy), putty (modostuc Gimod SRL, Pavia, Italy), yellow ochre bolus (CTS SRL, Bari, Italy), gelatin sheets (Dolciaria Pezzella SRL, Naples, Italy), 22 K gold leaf, 917/1000, dim. 8 × 8 cm (Aurum SAS gilding products, Bologna, Italy).

## 4. Results

### 4.1. Study and Characterization of Materials

**Figure 4:**
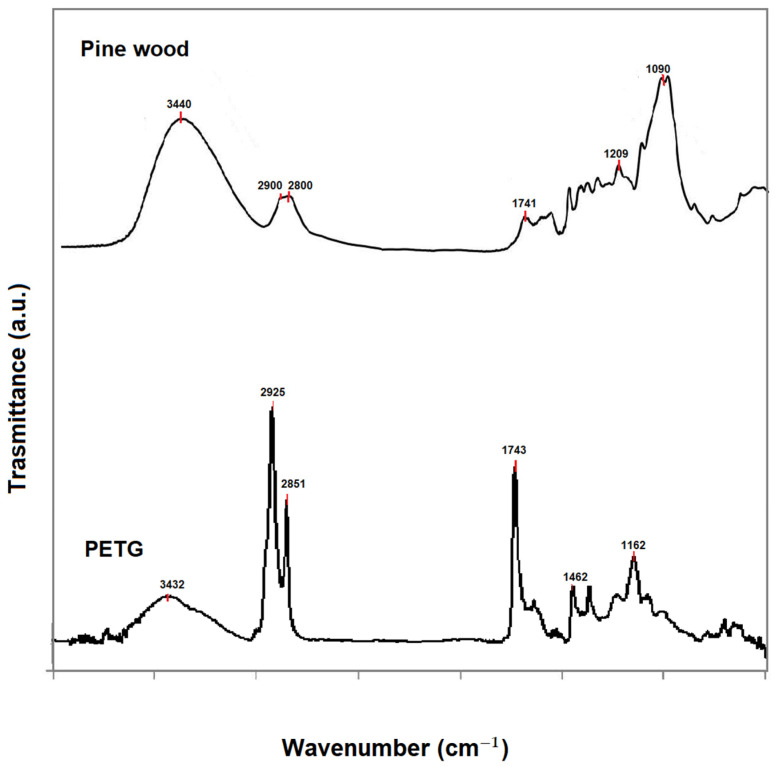
FTIR spectra of pine wood and PETG, and main infrared bands.

The chemical characterization of the neat materials used, pine wood and PETG, was carried out by identifying the main molecular groups using FTIR spectroscopy (Figure 4). The FTIR spectra of wood samples show infrared bands at 3440, 2900, and 2800 cm^−1^ associated with O-H and C-H groups; characteristic peaks in the fingerprint region at 1509 cm^−1^ (C-C stretching of the aromatic ring) and at 1209 cm^−1^ (C-O stretching) are associated with lignin, while at 1741 cm^−1^ (C-O stretching), 1640 cm^−1^ (C-O group), and 1090 cm^−1^ (C-O stretching) are associated with polysaccharides (hemicellulose and celluloses) [25,26,27]. The FTIR spectra of PETG show infrared peaks at 3432 cm^−1^ (O-H group), 2925 and 2851 cm^−1^ (C-H stretching), 1743 cm^−1^ (C-O ester group), 1462 cm^−1^ (C-C stretching); 1162 cm^−1^ (ester groups), 1091 cm^−1^ (C-H bends) [28]. The spectroscopic data agree with the literature [25,26,28]. In pine wood, no peaks associated with additives, fats, or impregnants are visible, but only the bands related to the main wood components: cellulose, hemicellulose, and lignin. Furthermore, the bands at 3400 cm^−1^ related to the stretching vibrations of the hydroxyl groups are different in the FTIR spectra of the two materials [29]. It is known in the literature that the position and shape of the OH stretching band can change in the specimens, and this will be positioned at about 3350 cm^−1^ for water chemically bound to the material and 3290 cm^−1^ for water absorbed from the external environment [29]. The size of the bands in the two samples results from this phenomenon, and the fact that the band at 3400 cm^−1^ in pine wood is greater than that in PETG indicates its greater hygroscopicity.

Pine wood and PETG test samples, produced as previously described, were subjected to scientific analysis before and after artificial aging in a climate chamber in order to compare the behavior of the two materials and their durability. The average results and relative standard deviations for each material are shown in Table 1.

**Figure 5:**
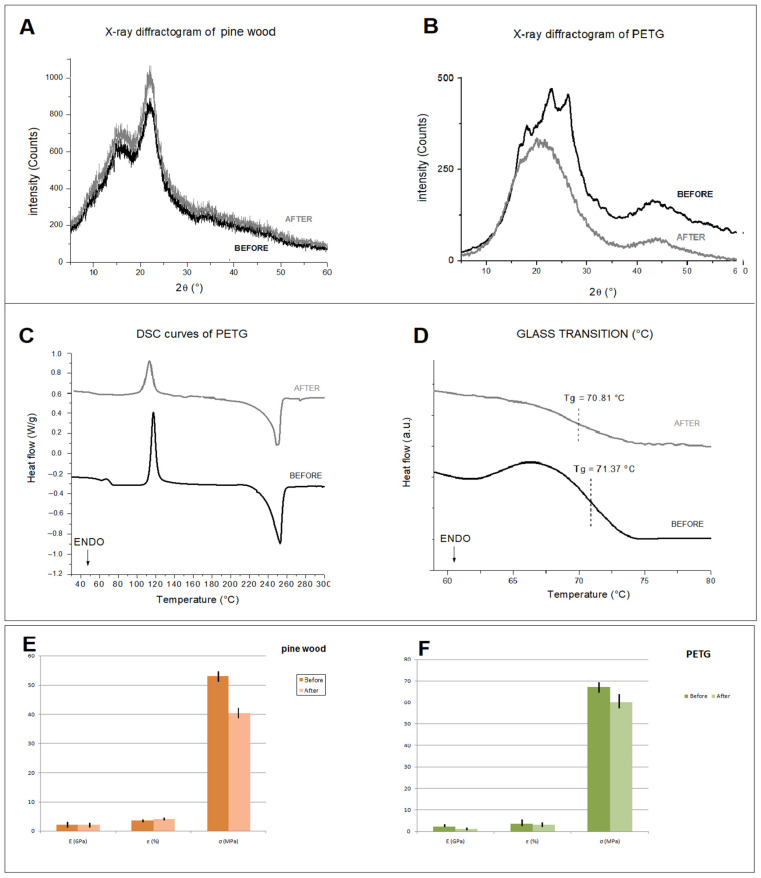
X-ray diffractogram of pine wood (**A**) and PETG (**B**) before and after aging; DSC curves of PETG (**C**) and glass transition temperature (**D**) before and after aging; flexural properties of pine wood (**E**) and PETG (**F**) before and after aging.

The artificial aging process had no significant effect on the size of the inspected samples. Instead, very small variations in weight were detected (Table 1). PETG preserves a good water repellency after artificial aging [30]; the contact angle value after aging is in fact 95.96 ± 5.56°, higher than the 90° limit conventionally used to distinguish a hydrophobic from a hydrophilic material [30]. In addition, it does not undergo appreciable color variations. These results agree with the literature, which reveals only a slight difference in brightness [9,24,31]. In contrast, pine wood shows a greater variation in the colorimetric coordinates L*, a*, b*, with a ∆E greater than five (Table 1). In fact, it is well known that wood is susceptible to photochemical degradation caused by exposure to light radiation, mainly due to lignin being a component [32]. Greater changes in thermal and mechanical properties are evident in the inspected samples (Figure 5). In the X-ray diffractogram of the wood sample (Figure 5A), the diffraction peaks at 15.3° and 22.2°, together with an amorphous background band, are evident; they originate from the crystalline and amorphous regions of the cellulose. Specifically, the first peak is assigned to the crystalline plane (101), while the second peak is assigned to the crystalline plane (002) [4]. The effect of artificial aging on the X-ray diffractograms of pine wood can be observed from the peak height at 22.4°, which increased significantly, in contrast to the peak height at 15.5° remaining unchanged (Figure 5A). These data are indicative of an increase in the crystallinity of the samples, as aging reduces the amorphous fractions of the wood [26]. The X-ray diffractogram of PETG (Figure 5B) shows the presence of peaks at 17.9°, 22.8°, and 26.1°, corresponding to crystal planes with Miller indexes of (010), (110), and (100), respectively [33]. As a result of artificial aging, an increase in the amorphous phase of PETG is observed, with a consequent decrease in the crystalline phase (Figure 5B). PETG neat shows a glass transition temperature (T_g_) of about 70.8 ± 0.5 °C (Figure 5C,D), calculated using the inflection point method and comparable with the reference literature [22,28]. After aging in a climate chamber, T_g_ of PETG is slightly reduced, indicating a decrease in the degree of crystallinity (Table 1, Figure 5C,D), which was previously indicated by XRD analysis. Accelerated aging can usually cause depolymerization of polymers, resulting in a less ordered structure [34]. This phenomenon involves the decomposition of the polymer into low molecular weight molecules, which occurs spontaneously through a natural aging process or, as in our artificial study, due to the influence of temperature and humidity [34,35]. Mechanical test data show different bending behaviors in the two materials (Figure 5E,F). This difference is accentuated by artificial aging and is more evident in pine wood. In particular, while the flexural modulus of elasticity (GPa) and the flexural stress (MPa) of both materials decrease after aging, contrastingly flexural strain (%) increases in pine wood (Figure 5E).

A general comparison of the morphological structural features of the two materials subjected to artificial aging shows a higher durability of PETG compared to pine wood, as was also reported in the literature [22,24,32,34,36]. However, to be exhaustive, the comparison must be applied to the specific case study analyzed and, thus, to the reproduction of the same column. In fact, although both materials are subject to a decrease in crystallinity and mechanical performance with aging, these characteristics are less evident in PETG, which preserves the water-repellent and colorimetric properties that are of fundamental importance to the preservation of the structural and chromatic properties of the columns and tabernacle. Furthermore, the increase in bending deformation manifested by pine wood with aging could cause greater instances of discontinuity surfaces in the multilayer system of the column (pictorial layers/gold leaf/stucco/support) and, consequently, more problems over time due to the detachment of the pictorial layers and the formation of lacunae on the column. As a result, PETG’s specific properties of good resistance and stability are more suitable for column reproduction for conservation purposes.

### 4.2. Replica of the Column by FFF Printing and CNC Milling

**Figure 6:**
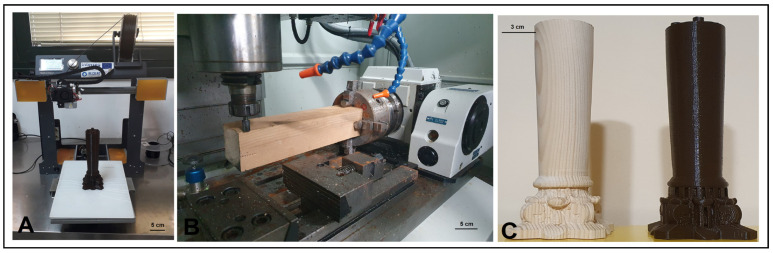
PETG column printing with BQ HEPHESTOS 2 printer (**A**); pine wood column production with HURCO VM 10 (**B**); comparison between the two columns produced (**C**).

**Figure 7:**
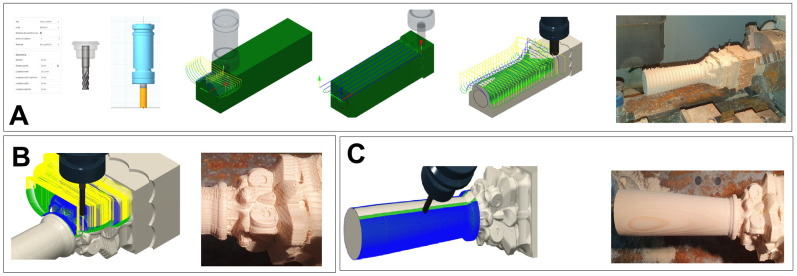
Initial roughing of the workpiece with the “parallel” function and a Ø 20 mm milling cutter (**A**); finishing operations on the capital (**B**); finishing operations on the lower truncated conical part of the column with the “rotary linear multi-axis” function (**C**).

A replica of the column was reproduced in PETG using the FFF technique (Figure 6A,C). Specifically, the printing strategy used facilitated the positioning of the object on the printer’s plate, eliminating the use of supports (thus with less waste of raw material). Furthermore, it allowed for layers perpendicular to the height of the column, enabling, during the final restoration phase, a good grip on the layers applied by the restorer to recreate the original texture. The following process parameters were used: nozzle 0.4 mm, infill 20%, extrusion temperature of 220 °C, plate temperature 50 °C, printing speed 60 mm/s, and layer height 0.2 mm. All details of the 3D printing can be found in our previous work [22]. For the reproduction in pine wood of the column by means of CNC milling (Figure 6B,C), the machine was first initialized (including warm-up and calibration phases), and the tools were assembled and set up (selection of tool position, assembly of the spindle tool holder collet, assignment of the identification number and probing cycle). Subsequent operations involved the assembly of the blank on the four-jaw chuck (considering a length of the blank greater than the actual length of the workpiece) and the setup of the workpiece zero, the position of which must coincide with the choice made during the design phase on the CAM software (Fusion 360 software (Autodesk, San Rafael, CA, USA)). Once the CNC machine setup was complete, the G-code file was uploaded. Due to the non-uniformity of the surface and section of the wood piece used, an initial roughing/finishing of the workpiece was carried out using the “parallel” function and a Ø 20 mm milling cutter (Figure 7A). The passes were carried out parallel to the plane selected, and the tool path followed the surface in the Z direction. Then, the workpiece was blocked in the chuck, placing the jaws on the processed area. The processing was continued by always using the “parallel” function and the following parameters: a Z step of 10 mm, a side step of 8 mm, a cutting feed rate of 1300 mm/min, a spindle speed of 5000 rpm, and an overmetal value of 5 mm. The milling cutter used was similar to the previous process. By contrast, the second processing was undertaken using the “parallel” function by a single pass, a side step of 6 mm, and the value of the supermetal reduced to 1 mm (Figure 7A). Finally, to reduce the average roughness, the third processing was carried out using a Ø 5 mm milling cutter, a lateral step value of 1 mm, and an oversize of 0.5 mm. After finishing the roughing operations, workpiece finishing was carried out. Initially, the capital was processed with a Ø 5 mm and a 0.25 mm lateral step milling cutter, obtaining an average ridge height value of 0.032 mm. Subsequently, the finishing of the capital was completed by using a Ø 1 mm cutter and a lateral step value of 0.1 mm (Figure 7B). The last operations involved finishing the truncated conical lower part of the column (Figure 7C). In this case, the “linear rotary multi-axis” function was chosen (lateral step equal to 1 degree and cutter Ø 5 mm). Partial truncations (90 degrees apart) were made using the “2D contouring” function (three passes with a Z step of 5 mm) and, finally, a manual truncation of the finished product.

### 4.3. CNC Milling versus FFF: The Case Study of the Missing Column of the Tabernacle

A general comparison between the advantages and disadvantages of the two analytical techniques investigated, CNC milling and FFF printing, is given as Supplementary Material S1.

In this section, the authors examine the same parameters for each technique, analyzing them according to the specific application: the reproduction of the missing columns of the 17th-century polychrome wooden tabernacle. There are, in fact, a lot of overlaps concerning the range of different possible applications. It is well known in the literature that CNC milling represents the best manufacturing solution for producing strong, accurate, and heat-resistant objects. However, FFF could be more suitable for different application fields. In this paper, we focus on a cultural heritage conservation application. We compare the different design and process steps required by both CNC milling and a low-cost FFF machine to produce the same column of a 17th-century polychrome wooden tabernacle, evidencing the advantages and disadvantages of the two building methodologies used for the same application. It is important to underline that the same column was printed using the FFF method with PETG and in the CNC milling machine, by using the same wood that the original tabernacle was made from.

The comparison of the properties of the two materials and the analysis of the accuracy of the models produced have been reported in previous Section 4.1 and Section 4.2. Here, the construction time, material waste, and total production cost in both cases were calculated and summarized in the following tables.

The time required for each building step necessitated by the two different technologies was determined by the laboratory technician using the information provided by the machines’ software, and it was also recorded using the stopwatch. The results obtained are reported in Table 2.

The calculation reported in Table 2 suggests that, for this specific application, CNC milling is more rapid than FFF according to the general considerations, as previously reported.

Table 3 reports a comparison of the costs required by the two building methodologies.

Specifically, Table 3 shows the material cost, operator cost, and tooling cost for both FFF and CNC milling. The material costs were determined by using the costs of materials reported on the technical data sheet, considering the amount of each material required by both FFF and CNC milling techniques to produce the same object. In particular, 210 g of PETG and 2400 g of pine wood were required by FFF and CNC milling, respectively. The operator cost for both the CNC and FFF was given by the lab technician, considering the unit cost of the operator and the total time reported in Table 2, without the duration of the building process (i.e., 15 h for FFF and 5 h for CNC milling). The tooling cost of each machine was determined by considering the price of each machine. In particular, the cost of FFF is EUR 1000, while the cost of CNC milling is EUR 11,0000. As a consequence of the data reported in Table 3, it is evident that the total production costs of CNC milling are a great deal larger than those of FFF. Finally, the mass of wood and PETG waste produced during the CNC milling and FFF process were calculated. In the case of FFF, the mass of waste is negligible since it was not necessary to produce supports, and, hence, no waste materials were obtained at the end of the 3D printing process. The calculation of wood waste mass (measured in grams) produced by the CNC milling process is reported as follows:

Wood waste mass = mass of the neat wood stock − mass of the produced column

The mass of the neat wood stock was measured by weighing the piece before using it; it is equal to 2374 g. The mass of the produced column was also determined by weighing it; it is equal to 240 g. It is, thus, evident that the CNC milling process produces a very large amount of waste material (about 2134 g).

Finally, Table 4 summarizes all the data collected by comparing the two techniques for the production of the column of the tabernacle.

### 4.4. Restoration

**Figure 8:**
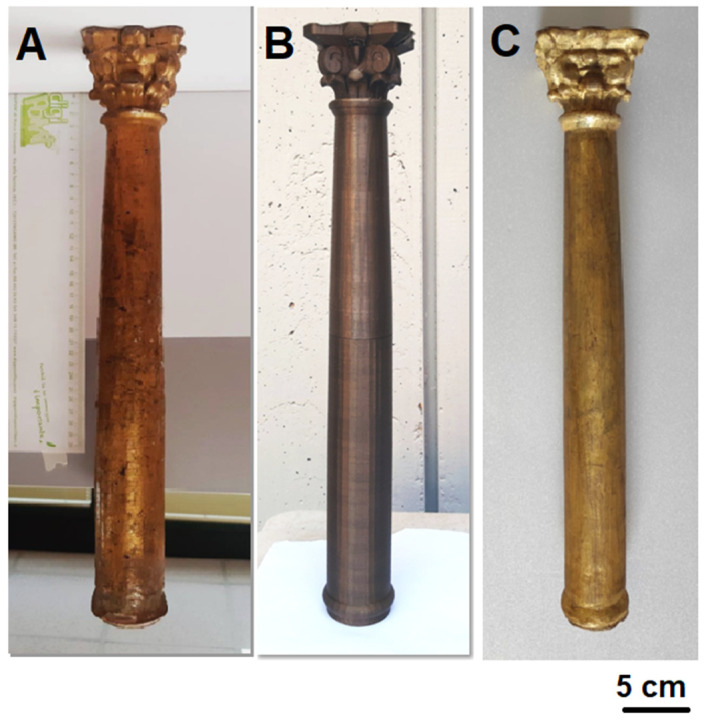
Original column (**A**); PETG printed column replica (**B**); and PETG column after restoration (**C**).

Based on the results obtained from the materials’ characterization and mainly from the comparison of the two production methods used (FFF and CNC milling), which showed the greater suitability of FFF as a technique to produce the missing column, the final restoration operations were carried out by the restorer exclusively on the PETG column, as reported: preparation of the substrate with Bologna plaster and rabbit glue, modeling of the stucco with a scalpel, application of red bolus, application of gold leaf with isinglass, and burnishing (Figure 8C). The success of the restoration is unmistakably shown by the images in Figure 8, clearly demonstrating the suitability of FFF techniques for this specific application, as well as the suitability of PETG for this conservative restoration.

## 5. Conclusions

In this paper, one of the of four missing columns of a 17th-century polychrome wooden tabernacle was reproduced using two of the most popular subtractive and additive technologies—FFF and CNC milling—with the aim of selecting the most suitable machine for this specific application. The analysis of the durability of the different materials used in the two machines, European pine wood (for CNC milling) and PETG (for FFF) confirmed that the mechanical properties of both materials decrease after accelerated aging weathering. However, PETG’s specific features of higher mechanical and wet resistance, stability and preservation of chromatic properties made it more suitable than pine wood for column reproduction for conservation purposes. This preliminary result was not a sufficient basis on which to select one of the two techniques, basing the decision only on the performances of the used material. For this reason, a deeper analysis based on a comparison between time, cost, and waste production of the two machines was performed. The calculations demonstrated that the building time of FFF is higher than that of CNC. However, the production costs and waste materials connected to the CNC process were much greater than those connected to FFF. Starting from this result, and considering that the different materials used for the realization of the two columns does not positively affect the steps of restoration, the authors selected the FFF process as the most suitable technique for producing the missing column. The 3D printed PETG column was, hence, successfully restored, clearly demonstrating the suitability of 3D printing for use in cultural heritage applications.

## Figures and Tables

**Table 1:** Comparison of surface, thermal, and mechanical properties of pine wood and PETG before and after artificial aging.

Sample	W (g)		CA (°)		∆E		Tg (°C)		E (GPa)		ε (%)		σ (MPa)	
	Before	After	Before	After	Before	After	Before	After	Before	After	Before	After	Before	After
Pine wood	2.10 ± 0.90	2.04 ± 0.07	/	/	/	9.03	/	/	2.22 ± 0.20	0.99 ± 0.13	3.71 ± 0.03	4.11 ± 0.02	53.01 ± 2.40	40.37 ± 1.89
PETG	3.92 ± 2.11	3.90 ± 1.08	98.36 ± 3.21	95.96 ± 5.56	/	2.60	70.81 ± 0.51	71.37 ± 0.73	2.35 ± 0.02	1.13 ± 0.01	3.61 ± 0.53	3.20 ± 0.64	67.10 ± 1.20	60.20 ± 2.11

**Table 2:** Comparison between time (h) of CNC milling and FFF for the production of a column of the 17th-century tabernacle.

Time (h)	FFF	CNC Milling
Design and Machining Strategies	2.0	4.0
Selection of Process Parameters	0.5	1.0
Simulation	1.0	1.5
Building Processing	15.0	5.0
Machine Cleaning	0	1.0
Total	18.5	12.5

**Table 3:** Comparison of costs of CNC milling and FFF for the production of a column of the 17th-century tabernacle.

Costs (Euro)	FFM	CNC Milling
	**PETG**	**Pine Wood Stock**
Material Cost	9.45	2.50
Operator Cost	375.00	125.00
Machine Cost	1000.00	110,000.00
Total	1384.45	110,127.5

**Table 4:** Comparison between costs of CNC milling and FFF for the production of a column of the 17th-century tabernacle.

	FFF	CNC Milling
	**PETG**	**Pine Wood Stock**
Time (h)	18.5	12.5
Costs (EUR)	1429	110.000
Material Wastage (g)	No wastage	2134

## Data Availability

Not applicable.

## Supplementary Materials

The following supporting information can be downloaded at: https://www.mdpi.com/article/10.3390/ma16083038/s1, Supplementary Materials S1: CNC milling versus FFF. References [1,9] are cited in the supplementary materials.
