# Supplementary material for: Fused Filament Fabrication and Computer Numerical Control Milling in Cultural Heritage Conservation
Source: Materials (Basel). 2023 Apr 12;16(8):3038. doi: 10.3390/ma16083038 (PMC10141658; doi:10.3390/ma16083038)
Supplement: Supplementary file 1 [file materials-16-03038-s001.zip › materials-2322792-supplementary.pdf]

Supplementari materials S1

CNC milling versus FFF

In this paragraph a comparison between the seleted CNC milling and FFF tecniques is reported. In particular, general considerations about the really important aspects of both processes are exposed (Table 1). While, the same analysis concerning the specific case study of the restoration of an ancient 17th-tabernacle is illustrated in the manuscript.

Process

CNC milling is a subtractive technique, differently from additive manufacturing method, used in FFF. CNC milling machines take a block of solid material and use sharp rotating tools or cutters to cut off each section that are not necessary. Instead, FFF allows the construction of a solid object by working on overlapping layers (layer manufacturing), starting from its CAD model.

Software

CNC mills are computer-controlled. The computer feeds them machinery-specific code that regulates the cutting tools (like the G-code used by 3D printers). The models for CNC mills are produced using 3D computer-aided manufacturing modeling software (CAM) and, recently, some CNC mills also accept STL and OBJ files (just like 3D printers).

Materials

CNC mills operate with several materials, like for example metal alloys, soft or hard wood, thermoplastics, acrylic, modeling foams, machining wax. Different cutting tools are necessary for different materials, but the tool-to-machine interfaces are generally identical, and, thus, the tools can simply be replaced. For this reason, CNC mill can be used to fabricate prototypes in the same material that will be used to produce final object. On the other hand, FFF is generally limited to a small number of thermoplastic polymers, such as PLA, ABS, nylon, PET. Some fillers, such as ceramics, wood, metal, can be added to the thermoplastics matrices in order to improve their properties [1]. However, the workpieces made by FFF will not exhibit high thermo-mechanical properties and durability, comparable to that of the same workpieces obtained by cutting from a block of metal or wood, with a CNC milling.

Accuracy

CNC mills present position accuracies of about 0.025 mm and tolerances of 0.0127 mm. It is well known that there are specific FFF machines able to guarantee even resolutions of 0.025 mm. However, the material generally used in a CNC mill lets considerably superior precision than that reached by using a thermoplastic material, like in a FFF technique.

Time

As previously explained, CNC mills and FFF usually work with different materials to produce different workpieces. Consequently, it is not easy to compare the building time required by the two techniques to produce the same

object. However, FFF frequently takes hours to complete the building, while CNC milling normally does not take more than an hour to produce a piece with analogous size and complexity.

Noise

As a function of the material, CNC milling can be tremendously noisy. Cutting metal or wood working with a huge-diameter tool can be ear-crashing. Even cutting wax prototypes, the noise produced by a CNC mill is strongly detectable. On the other hand, FFF printer produces a very slight noise.

Vibrations

Working on a metal or wood block, a CNC mill severely vibrates. On the contrary, a desktop FFF, generally, does not produce any vibration.

Cleaning

Because of the subtractive process of the CNC milling (i.e., cutting away material using a rotating tool), a lot of material is rushed away. Furthermore, not all CNC mills are fully enclosed when working on a block of material, and hence, some things can be very messy, requiring to clean up the mess inside, once the workpiece is finished. On the contrary, FFF is not messy by design. Only, when something goes wrong, it is necessary to remove thermoplastics from the 3D printed model. But this is still very limited compared to cleaning up after CNC milling. Some postprocessing may be required both after 3D printing and CNC milling: grinding and sanding. But we wouldn’t call that messy.

Waste materials

FFF technologies produce a small amount of waste [1]. The 3D printing process, in fact, only involves the material necessary for building the 3D model. On the other hand, CNC milling technology requires a block of material that has at minimum the size of the workpiece. For this reason, a great amount of material must be removed and frequently cannot be recycled.

Costs

Whole production costs are a crucial aspect of the manufacturing process 3 D models. There are a lot of costs related to the specific process. The most important are operator cost, material cost and tooling cost. Operator costs depend on the skill level necessary for machining. Material costs increase as mechanical properties of the material increase. Tooling cost depends on what kind of material is used for the cutting tool and how much it wears out while making the cut on the material to be machined [2]. All the aspects that have to be considered in the comparison between CNC milling and FFF are reported in Table 1.

Table S1. Diagram summarizing all aspects analyzed to compare the two technique.

Advantages/disadvantages	CNC milling	FFF
Noise	↑	↓
Vibrations	↑	↓
Cleaning	↑	↓
Waste materials	↑	↓
Process	_	_
Software	_	_
Materials	↑	↓
Accuracy	↑	↓
Costs	↑	↓
Time	↓	↑

References

1. Fico, D.; Rizzo, D.; Casciaro, R.; Esposito Corcione, C. A Review of Polymer-Based Materials for Fused Filament Fabrication (FFF): Focus on Sustainability and Recycled Materials. *Polymers* **2022**, *14*, 465, https://doi.org/10.3390/polym14030465.
2. Pereira, T.; Kennedy, J. V.; Potgieter, J. A Comparison of Traditional Manufacturing vs Additive Manufacturing, the Best Method for the Job. *Procedia Manuf.* **2019**, *30*, 11–18, https://doi.org/10.1016/j.promfg.2019.02.003.
